# *Bacillus subtilis* Spore-Trained Dendritic Cells Enhance the Generation of Memory T Cells via ICAM1

**DOI:** 10.3390/cells10092267

**Published:** 2021-08-31

**Authors:** Jian Lin, Lulu Huang, Yuchen Li, Penghao Zhang, Qinghua Yu, Qian Yang

**Affiliations:** 1 College of Life Sciences, Nanjing Agricultural University, Weigang 1, Nanjing 210095, China; linjian@njau.edu.cn; 2 MOE Joint International Research Laboratory of Animal Health and Food Safety, College of Veterinary Medicine, Nanjing Agricultural University, Weigang 1, Nanjing 210095, China; huanglulu900601@163.com (L.H.); yuchengli0016@126.com (Y.L.); 2018107022@njau.edu.cn (P.Z.); yuqinghua1981@163.com (Q.Y.)

**Keywords:** *Bacillus subtilis* spore, intestinal-resident memory T cells, dendritic cell, ICAM1, Acrp30

## Abstract

Immunological memory is a cardinal feature of the immune system. The intestinal mucosa is the primary exposure and entry site of infectious organisms. For an effective and long-lasting safeguard, a robust immune memory system is required, especially by the mucosal immunity. It is well known that tissue-resident memory T cells (Trms) provide a first response against infections reencountered at mucosal tissues surfaces, where they accelerate pathogen clearance. However, their function in intestinal immunization remains to be investigated. Here, we report enhanced local mucosal and systemic immune responses through oral administration of H9N2 influenza whole inactivated virus (H9N2 WIV) plus *Bacillus subtilis* spores. Subsequently, H9N2 WIV plus spores led to the generation of CD103^+^ CD69^+^ Trms, which were independent of circulating T cells during the immune period. Meanwhile, we also found that *Bacillus subtilis* spores could stimulate Acrp30 expression in 3T3-L1 adipocytes. Moreover, spore-stimulated adipocyte supernatant also upregulated the expression of intercellular adhesion molecule-1 (ICAM1) in dendritic cells (DCs). Furthermore, the proportion of HA-tetramer^+^ cells was severely curtailed upon suppressed ICAM1 expression, which also depended on HA-loaded DCs. Taken together, our data demonstrated that spore-promoted H9N2 WIV induced an immune response by enhancing Trms populations, which were associated with the activation of ICAM1 in DCs.

## 1. Introduction

H9N2 subtype avian influenza virus (AIV), a low-pathogenicity but highly endemic AIV, has become a significant threat to humans and animals [1,2]. H9N2 AIV attacks and invades the host from the mucosa of the respiratory tract, and then spreads via fecal–oral transmission [3,4]. Hence, efficient mucosal immunity can effectively block the entry of the infectious organism, making mucosal vaccination a prominent strategy to prevent pathogenesis. However, mucosal immunization with inactivated virus alone has been demonstrated to be not enough to strongly induce a mucosal immune response [5,6]. Thus, the use of adjuvants to improve vaccine potency has become critical for mucosal vaccine development. *Bacillus subtilis* spores, acting as adjuvants, can strongly induce immune responses against pathogens, especially by modulating intestinal mucosal immunity by evoking tissue-resident memory T cells (Trms) [7,8,9].

Previous studies found that *Bacillus subtilis* spores can stimulate the secretion of cytokines during innate immune signaling, which is indispensable for an efficient induction of adaptive immune responses during primary immunization [10]. A recent study found that mucosal immunization with Spore-FP1 increased the levels of CD69^+^ CD103^+^ Trms in lung parenchyma [11]. It is well known that mucosal vaccine-mediated intestinal T-cell responses reveal a requirement for the addition of adjuvants for evoking a robust Trm response [12]. Moreover, Trms from the intestinal mucosa mediate rapid clearance of and heterosubtypic protection against secondary AIV infections in mice [13,14]. Our previous study also confirmed that *Bacillus subtilis* spores, facilitating mucosal delivery of porcine epidemic diarrhea whole inactivated virus (PEDV WIV), could regulate memory T cells in the intestine of piglets [15]. However, the mechanism underlying *Bacillus subtilis* spores inducing the proliferation of effective memory T cells in intestine is still unknown. Dendritic cells (DCs), the most important submucosal antigen-presenting cells (APCs), are mainly responsible for the connection between innate and acquired immunity [16,17]. *Bacillus subtilis* spores have been demonstrated to be a possible alternative oral adjuvant evoking T-cell memory responses following conjugation to a carrier protein [15]. The aim of present study was to analyze whether *Bacillus subtilis* spore-activated Trms can be regulated by dendritic cells. In addition, intercellular adhesion molecule-1 (ICAM1) is critical for establishing memory T-cell populations following acute infection [18]. For instance, a substantial number of liver-resident memory T cells are regulated by the LFA-1–ICAM1 interaction following lymphocytic choriomeningitis virus (LCMV) immunization [19]. Furthermore, lipid metabolism-related molecules play an important role in regulating ICAM1 expression [20]. Hence, our study aimed at assessing the mechanism underlying *Bacillus subtilis* spore induce memory T-cell formation through the activation of ICAM1 expressed by DCs.

## 2. Methods

### 2.1. Animals and Ethics Statement

This study was approved by the Ethics Committee of Animal Experiments center of Nanjing Agricultural University. All animal studies were approved by the Institutional Animal Care and Use Committee of Nanjing Agricultural University (SYXK-2017-0007) and followed the National Institutes of Health guidelines for the performance of animal experiments. Specific pathogen-free C57BL/6 (4 to 6 weeks) and BALB/c (6 to 8 weeks) mice were obtained from Comparative Medical Center of Yangzhou University (Jiangsu, China). All animals were maintained at an animal facility under pathogen-free conditions. 

### 2.2. Vaccine Preparation

The influenza A/Duck/Nan Jing/01/1999 H9N2 virus was generously provided by the Jiangsu Academy of Agricultural Sciences. The H9N2 virus was purified using a discontinuous sucrose density gradient. H9N2 WIV is normally inactivated via incubation at 56 °C for 30 min to achieve a complete loss of infectivity. The *Bacillus subtilis* SQR9 strain was kindly supplied by Professor Shen of Nanjing Agricultural University [21]. 

### 2.3. Immunogenicity Study

Immunological memory is a critical adaptation of the immune system that mediates more rapid and effective responses to previously encountered antigens. We performed preliminary experiments and found that the effectiveness of immunizations was optimal when the mice were administered orally twice with an interval of 7 days. We, therefore, selected primary and second immunization at 0 days and 7 days. We found that the antibody titers induced by *B. subtilis* spores significantly increased from days 7 to 35 in mice but decreased on days 35 and 42. Boost immunization was selected on day 42. On this basis, 6 week old BALB/c mice were orally immunized with H9N2 WIV (20 μg) alone or in combination with spore (10^7^ CFU) three times (at 0, 7, and 42 days) by gavage. The mice were euthanized, and samples were collected at 1 week intervals after primary immunization. The levels of specific IgA in intestinal lavage fluid and of specific IgG, IgG1, and IgG2a in serum were detected using ELISA. In brief, a plate was coated with H9N2 WIV (2 μg/mL) antigens overnight, which were then blocked for 2 h with PBST containing 3% BSA. Intestinal lavage fluid and serum were diluted in PBS with 0.1% BSA and added to the plate in triplicate for 1.5 h at 37 °C. After five washes, HRP-conjugated rabbit anti-mouse IgG was incubated on the plate for 1 h. OD_450_ values were read. A hemagglutination inhibition (HI) test was performed according to a previously described procedure [22]. Meanwhile, the proliferative response was detected using CCK-8 assays according to the manufacturer’s instructions (BOSTER, Wuhan, China).

### 2.4. Intestinal Mucosa-Associated Lymphocyte (IMAL) Isolation

IMALs were isolated as described previously [23]. In brief, the intestine was opened longitudinally after the removal of residual mesenteric fat tissue. The tissue was then dissected into pieces and thoroughly washed with ice-cold PBS followed by digestion with 0.5 mg/mL collagenase D (Sigma, St. Louis, MO, USA), 0.5 mg/mL DNase I (Roche, Man-nheim, Germany), and 50 U/mL Dispase^®^ I enzyme (Sigma, St. Louis, MO, USA)in Dulbecco’s phosphate-buffered saline (DPBS) containing 5 mM EDTA, 4% fetal calf serum, and 100 μg/mL penicillin/streptomycin for 30 min at 37 °C with slow rotation (100 rpm). After incubation, cells were collected, filtered through a 70 μm strainer (BD Biosciences, San Jose, CA, USA), and washed once with cold RPMI-1640. Then, the cells were resuspended in 6 mL of a 30% fraction of Percoll continuous density gradient and overlaid on 6 mL of the 70% fraction in a 15 mL Falcon tube. Percoll gradient separation was performed by centrifugation at 300× *g* for 20 min. IMALs were collected at the interphase of the Percoll gradient, washed once, and resuspended in cold RPMI-1640 with 5% FBS. The cells were used immediately for experiments. All other cell cultural reagents were from Thermo (Thermo Fisher Scientific, Waltham, MA, USA).

### 2.5. Flow Cytometry and Cell Sorting

For most experiments, cells were first incubated with an Fc receptor blocker (1:20 dilution; eBioscience). For surface staining, cells were then stained with a mix of fluorescent antibodies in flow cytometry buffer for 30 min at 4 °C according to the manufacturer’s guidelines. For Trms flow cytometry, cells were separately stained with CD3-APC (145-2C11, eBioscience, San Diego, CA, USA), CD103-FITC (2E7, eBioscience, San Diego, CA, USA), and CD69-PE (H1.2F3, eBioscience, San Diego, CA, USA). For Tcms in blood, cells in 50 μL of blood were stained with CD3-percp-cy5.5 (1452C11, Miltenyi Biotec, Bergisch Gladbach, Germany), CD62L-APC (REA828, Miltenyi Biotec, Bergisch Gladbach, Germany) and CCR7-PE (REA685, Miltenyi Biotec, Bergisch Gladbach, Germany). Then, the whole blood was filtered through a 70 µm cell strainer, and the suspensions were incubated with an ammonium chloride potassium lysis buffer for 20 min at RT. For intracellular staining, the cells were incubated with 50 ng/mL phorbol myristate acetate (PMA; Sigma, St. Louis, MO, USA), 750 ng/mL ionomycin (Sigma, St. Louis, MO, USA), and 10 μg/mL brefeldin A (Invitrogen) in a cell culture incubator at 37 °C for 5 h. After surface staining, the cells were resuspended in fixation and permeabilization solution (BD Biosciences, San Jose, CA, USA) for 45 min at 4 °C. Consistently with previous reports, the signature cytokines, interleukin (IL)-4 and IFN-γ, were measured using a BD flow cytometry Verse and analyzed with FlowJo v.10 (Tree Star, Inc., Ashland, OR, USA).

For the lymphocyte enrichment assay, CD3 T cells were purified by negative selection reagent (Mouse T Lymphocyte Enrichment Set-DM; BD Biosciences, San Jose, CA, USA) similarly to previously described methods [24]. Briefly, single-cell suspensions of lymph node (LN) or enteric cells were incubated with the following dilutions provided for each: for staining, a 100 μL volume of antibody cocktail was used per tissue sample from one mouse. Resuspended cells were incubated in antibody cocktail for 15–30 min at 4 °C in the dark. After washing of cells with 10 mL of PBS, the mixture was passed over a magnet following the manufacturer’s instructions. The purity of the flowthrough fraction was routinely >90%.

### 2.6. Adipocyte Differentiation in Cell Culture

Mouse 3T3-L1 preadipocyte cells were cultured and differentiated as previously described [25]. Briefly, 3T3-L1 cells were grown in regular medium (high-glucose Dulbecco’s minimum essential medium (DMEM) supplemented with 10% FBS containing 1% penicillin and streptomycin). About 2 × 10^5^ cells were seeded on 12-well plates and grown to full confluence for 4 days. The cells were then subjected to the first differentiation medium (DMEM supplemented with 10% FBS, 0.5 mM 3-isobutyl-1-methylxanthine, 1 µM dexamethasone, and 10 µg/mL insulin) starting on day 0 after confluence. After 2 days of induction, the medium was replaced with only insulin in DMEM with 10% FBS for an additional 2 days. Then, 2 days later, the cells were grown in regular medium for an additional 8 days, and the medium was replaced every 2 days. Isobutyl-1-methylxanthine, dexamethasone, and insulin were obtained from Sigma-Aldrich. The 3T3-L1 cells were obtained from Professor Yang Xiaojing of the Nanjing Agriculture University. In this study, the medium was taken from 3T3-L1 adipocytes treated with 10^6^ and 10^7^ CFU/mL of spores for 24 h. Before adding to the DCs, the medium was filtered with a 0.2 μm membrane. Then, the supernatant from the adipocyte cell culture was added to the DCs for another 24 h. All other cell cultural reagents were from Thermo (Thermo Fisher Scientific, Waltham, MA, USA)

### 2.7. FTY720 Treatments and Tetramer Staining

To inhibit the circulation of memory T cells, 1 mg/kg of FTY720 (Sigma, St. Louis, MO, USA) in PBS was intraperitoneally (i.p.) administered daily for 10 days. In addition, to assess the protective efficacy of the vaccines, mice were immunized with the same vaccine. Intravascular staining was performed by injecting mice i.v. with FITC-conjugated anti-mouse CD45 antibody (5 μg) for 8–10 min before euthanasia (Beckman Coulter, Villepinte, France). After immunization and FTY720 treatment (Novartis Pharma AG, Basel, Switzerland), intestinal tissue was collected and collagenase-digested, and cells were isolated for flow cytometry analysis as described previously. The lymphocytes were isolated from the intestine of mice 6 weeks after immunization. Antigen-loaded DCs treated or not with ICAM1 inhibitor were incubated with lymphocytes isolated from immunized mouse intestine at 37 °C for 3 days. Collected cells were stained with anti-CD3 antibody for 20 min and with HA_518–526_^+^ tetramer for 1 h. Moreover, to assess whether HA-tetramer^+^-specific T cells were present in the intestine at 6 weeks after immunization, antigen-loaded DCs treated or not with ICAM1 inhibitor were incubated with IMALs isolated from immunized mice at 37 °C for 3 days.

### 2.8. DC/EC Coculture System 

Since C57BL/6 was mostly suitable mice used for isolated dendritic cells, we selected and isolated DCs from C57BL/6 C57BL/6 mice. DCs were generated from 4 to 6 week old C57BL/6 mice using our previous method [26]. Briefly, bone marrow was extracted from the tibias and femurs of C57BL/6 mice with RPMI-1640. Then, the cells were suspended in complete medium (RPMI-1640 supplemented with 10% heat-inactivated FBS, 1% PenStrep), supplemented with 10 ng/mL IL-4 and granulocyte-macrophage colony-stimulating factor (GM-CSF). After culture for approximately 60 h, the medium was gently discarded to detach nonadherent granulocytes. Then, clusters were harvested, and the adherent cells were removed after 5 days. Nonadherent cells were collected at 6 days and used in subsequent studies. Caco-2 cells were seeded on the upper side of ThinCert^TM^ membrane inserts (pore size, 3 μm) (Greiner Bio-One, Frickenhausen, Germany) in a 24-well plate and grown overnight. The cells were maintained for 6 to 10 days until a steady-state transepithelial electrical resistance of 300 Ω·cm^2^ was achieved. In the coculture system, the filters were turned upside down, and then DCs (5 × 10^5^ cells/mL) were cultured on the basolateral side of ECs referring to Caco-2 cells for 4 h to let the cells attach to the filter. The filters were then turned right side up and placed into 24-well plates. The cells were incubated for 24 h with spore (10^7^ CFU/mL) or lipopolysaccharide (LPS) (1 μg/mL) administered from the apical side. The filters and cells were then fixed with 4% paraformaldehyde (PFA) for 15 min and processed for confocal microscopy. In addition, DCs were collected for phenotype assays, and basolateral supernatants were collected for cytokine secretion assays.

### 2.9. Ligated Loop Experiments 

Mice were anesthetized with chloral hydrate (350 mg/kg body weight, intramuscular injection). The in situ ligated terminal jejunal and ileal ligated loops were injected with spores (10^8^ CFU/mL) or the same volume of PBS (0.01 M). Then the intestines were removed after 0.5 h, put in OCT (Tissue Freezing Medium, Sakura, Torrance, CA, USA), and cut into 8 μm for immunofluorescence assays, as described below.

### 2.10. Mouse Cytokine Array by a Proteome Profiler

Intestinal tissues from mice treated with PBS or spore for 7 days and 45 days were lysed with cell lysis buffer (R&D Systems, Minneapolis, MN, USA) supplemented with 1% 0.2 mM phenylmethylsulfonyl fluoride (PMSF) at 4 °C for 30 min. The protein concentration was detected with a protein BCA kit (Thermo Fisher Scientific, Waltham, MA, USA). Samples were analyzed with a mouse XL cytokine array kit (R&D Systems, Minneapolis, Minn., USA), according to the manufacturer’s instructions. Immunospots were captured with an Odyssey Fc Imager (LI-COR Biosciences, Lincoln, NE, USA) and analyzed with Image-J software (v1.45 NIH USA).

### 2.11. Histology and Immunohistochemistry

Immunohistochemistry detection was performed with the SABC kit (Boster Bioscience). The endogenous peroxidase activity was neutralized using 3% H_2_O_2_ at 37 °C for 30 min, and the sections were rinsed three times with PBS for 15 min after antigen retrieval was performed with citrate buffer (containing 2 mM citric acid and 10 mM trisodium citrate; pH 6.0). Tissue sections were incubated with primary antibodies against ICAM1 (1:200; Abcam) overnight at 4 °C. Subsequently, the sections were incubated with biotinylated goat anti-mouse IgG as the secondary antibody. After staining with DAB, images were captured using a digital camera (Leica-DM4000B).

### 2.12. Immunofluorescence (IF)

The fixed filters were permeabilized in 0.5% Triton X-100 in PBS for 5 min and blocked with 5% bovine serum albumin (BSA) in PBS for 2 h. Then, the filters were stained with primary antibodies Armenian hamster anti-CD11c (N418) and rabbit anti-ICAM1 (1A29, Abcam) overnight at 4 °C, followed by incubation with secondary antibodies for 2 h at room temperature. For the in vivo model, the cryosection was treated as described above. The filters were identified using confocal laser scanning microscopy (CLSM) (LSM 710; Zeiss, Oberkochen, Germany). Cross-sectional images were observed by *z*-axis views and analyzed using Zeiss ZEN 2012 and Adobe Photoshop CC (Adobe, San Jose, CA, USA).

### 2.13. Quantitative RT-PCR (qRT-PCR)

Total RNA from intestinal tissues was prepared using Trizol reagent (Takara, Kyoto, JPN) following the manufacturer’s guidelines and reverse-transcribed using a PrimeScript RT reagent Kit (Takara, Kyoto, JPN) according to the manufacturer’s instructions. QPCR was performed for triplicate samples using a SYBR Green qPCR Kit (Takara, Kyoto, JPN) by the Applied Biosystems™ QuantStudio™ 6 standard Real-Time PCR System (Thermo Fisher Scientific, Waltham, MA, USA). The housekeeping gene β-actin was routinely used as an internal control. The primers used in this study were as follows: for β-actin, 5′–AAGTGTGACGTTGACATCCG–3′, rev 5′–GATCCACATCTGCTGGAAG–3′; for ICAM1, 5′–TCACCAGGAATGTGTACCTGAC–3′, rev 5′–GGCTTGTCCCTTGAGTTTTATGG–3′. Primer used for IL15 were 5′–ACATCCATCTCGTGCTACTTGT–3′ and rev 5′–GCCTCTGTTTTAGGGAGACCT–3′. Primers used for TGF-β were 5′–CACCATCCATGACATGAACC–3′ and rev 5′–TCATGTTGGACAACTGCTCC–3′.

### 2.14. Western Blot Assay

The cells were lysed with RIPA buffer containing a 1% protease inhibitor cocktail on ice for 20 min. After removing debris by centrifugation at 4 °C, supernatant protein was collected, and the total concentration was determined using a BCA protein assay kit. Protein was separated by electrophoresis on 10% sodium-dodecyl sulfate polyacrylamide gels (SDS-PAGE) and transferred to a polyvinylidene difluoride (PVDF) membrane. Mouse anti-ICAM1 (1A29, Abcam, Cambridge, UK), anti-Acrp30 (PA1-054, Thermo Fisher Scientific, Waltham, MA, USA)), and anti-β-actin (4D3, Bioworld Technology, St. Louis Park, MN, USA) were used to assess ICAM1 and Acrp30 expression. Western blot images were visualized using an Image Reader Tanon-5200 imaging system.

### 2.15. Statistical Analysis

Results are shown as the mean ± SEM. Student’s *t*-test was employed to determine the difference between two groups, and one-way analysis of variance (ANOVA) with Dunnett’s test was performed using SPSS among multiple groups. In short, this test corresponds to an independent-sample *t* test, which is used when the normal distribution and variance uniformity cannot meet the requirements of a *t*-test. For multiple-group comparisons, we used one-way analysis of variance (ANOVA) with Dunnett’s test, which is similar to the Kruskal–Wallis method. Since our data were totally in accordance with a normal distribution, we chose the *t*-test and one-way ANOVA for our analysis. The statistical analysis was performed using FlowJo v10, Microsoft Excel 2010, and Graph Pad Prism 7 Software (La Jolla, CA, USA). The asterisks indicate significant differences between H9N2 WIV plus spore and H9N2 WIV; a *p*-value <0.05 was considered to be statistically significant (* *p* < 0.05, ** *p* < 0.01).

## 3. Results

### 3.1. Spore-Adjuvant H9N2 WIV Induces the Production of Specific Antibodies and Prompts Lymphocyte Proliferation 

**Figure 1:**
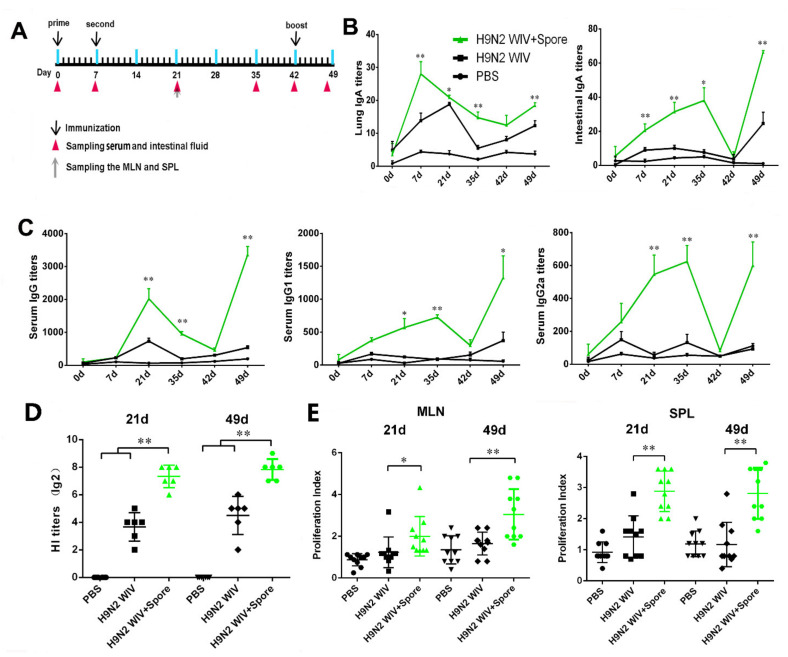
Spores promote H9N2 WIV-dependent production of antigen-specific antibodies. (**A**) Schematic of oral immunization and the sampling schedule of intestinal fluids, lung wash fluids, serum, mesenteric lymph nodes (MLNs), and spleen (SPL). H9N2 WIV (20 μg) and spores (10^8^ CFU/mL) were orally administered to each mouse. Primary and secondary immunizations were performed at 0 days and 7 days, respectively. Booster immunizations were administered at 42 days. The details of the immunization schedule indicate the timepoint of immunization (black arrows above the line). The sampling timepoints of the serum, intestinal fluid, and lung washing buffer are indicated by arrows below the line. (**B**,**C**) H9N2-specific IgA and IgG antibodies in mice after immunization were determined using ELISA. (**C**) Antigen-specific serum IgG titers, IgG1 titers, IgG2a titers, and mucosa IgA titers in intestinal wash and (**B**) lung wash fluids were detected at different timepoints. The asterisks indicate significant differences between H9N2 WIV plus spores and H9N2 WIV alone. (**D**) Hemagglutination inhibition (HI) titers were detected at 21 days and 49 days. The results are expressed as the mean ± SEM. (**E**) MLN and splenic lymphocytes from immunized mice were isolated and restimulated with H9N2 WIV (10 μg/mL) in vitro. The proliferative response was detected using a CCK8 assay. A *p*-value < 0.05 denotes a statistically significant difference (* *p* < 0.05, ** *p* < 0.01) (*n* = 6).

Oral immunization is beneficial for eliciting mucosal immune responses against pathogens that invade their host through a mucosal surface. However, inactivated vaccines, such as H9N2 WIV, have been deemed poorly efficient in inducing strong immunization. Thus, our study used spores as a mucosal adjuvant to enhance the mucosal immune response to the inactivated vaccine. Since mucosal immunization induces substantial IgA production, but weak IgG production, as shown in Figure 1A, we used a mucosal immunization strategy known as “prime-boost vaccination” and detected antibody levels after mucosal immunization with spores plus H9N2 WIV. Local secretion of IgA antibodies is the most important characteristic mediating oral adaptive immunity and mucosal protection. Firstly, we evaluated the IgA level and found that spore-promoted H9N2 WIV significantly enhanced IgA concentration in the lung wash and intestine after oral immunization of mice (Figure 1B). This result suggested that spores can act as a good mucosal adjuvant to induce mucosal IgA responses in the lower respiratory tract. Secondly, we found a trend indicating that spore plus H9N2 WIV could induce increased levels of H9N2-specific IgG in serum (Figure 1C). Further analysis revealed that the levels of both IgG1 and IgG2a induced by H9N2 WIV plus spores at 21 days, 35 days, and 49 days were significantly higher than the levels induced by H9N2 WIV alone (Figure 1C). Thirdly, we found that serum collected from different groups of mice at 21 days and 49 days showed a powerful ability to inhibit hemagglutination by 4-HA units of H9N2 compared with antigen alone (Figure 1D) Lastly, lymphocytes from the spleen and mesenteric lymph node (MLN) of mice 21 days and 49 days post immunization were restimulated with H9N2 WIV in vitro. We found that the proliferative index of MLN and splenic lymphocytes was markedly increased after treatment with spore plus H9N2 WIV with respect to antigen alone (Figure 1E) (*p* < 0.05), reflecting the effective induction of systemic and local immune responses in mice.

### 3.2. Spores Plus H9N2 WIV Induce CD69^+^ CD103^+^ Trms in Intestinal Tissue 

**Figure 2:**
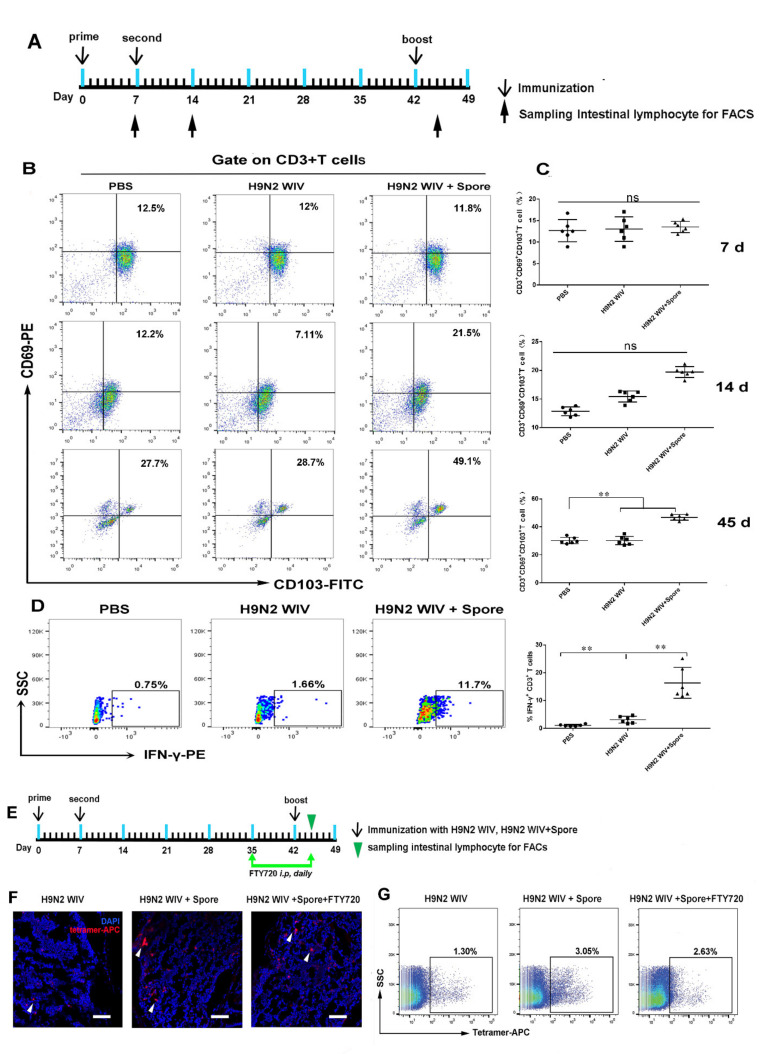
Evidence of HA-specific T cells in the intestinal tract after oral immunization. (**A**) Schematic experimental design applied to examine the frequency of Trms after oral immunization with different vaccines. (**B**,**C**) The frequency of Trms (CD3^+^ CD69^+^ CD103^+^) was detected in the intestinal mucosa 7 days, 14 days, and 45 days after priming immunization. A gating strategy was applied in this study to determine the memory-cell phenotype of CD3^+^ T cells from intestinal tissue according to the expression of CD69 and CD103. (**D**) IFN-γ expression in CD3^+^ T cells from immunized mice was detected by flow cytometry following restimulation with H9N2 WIV. Data are presented as the mean ± SEM (*n* = 6). (**E**) BALB/c mice were immunized as described previously, and lymphocytes from the intestine were analyzed by HA-specific tetramer staining at 45 days to detect the effect of FTY720 treatment on T cells. Five weeks after immunization, immunized mice were treated with 1 mg/kg of FTY720 i.p. daily for 10 days. (**F**,**G**) APC-tetramer^+^ T-cell populations were compared between immunized mice treated with H9N2 WIV plus spores and treated or not with FTY720, after analysis by confocal laser scanning microscopy (CLSM) (F) and FACS (G). Scale bar = 50 μm. Data are presented as the mean ± SEM (*n* = 6); ** *p* < 0.01.

The abovementioned mucosal immunization not only led to robust antibody responses, but also generated long-lasting immunological memory T cells after 49 days. As is known, tissue-resident memory T cells (Trms) are an important first line of defense against infections [27]. A recent study demonstrated that Trms can be found in several tissues, including the intestinal and lung mucosa, and they can be distinguished from other memory T cells by CD103 and CD69. Therefore, we investigated whether spores plus H9N2 WIV could induce Trms formation in vivo. To verify this hypothesis, intestinal tissues were harvested from immunized animals, and CD3-positive cells were then assessed for the population of Trms by examining the expression of the tissue retention markers CD69 and CD103 at 7 days, 14 days, and 45 days (Figure 2A). We found no significant difference of CD69^+^ CD103^+^ cells population among PBS, H9N2, and H9N2 plus spore-treated groups at 7 and 14 days (Figure 2B,C). However, the population of CD69^+^ CD103^+^ Trms in 45 days (49.1%) after spores plus H9N2 WIV immunization was significantly higher than that in PBS- (27.7%) or H9N2-treated (28.7%) groups (Figure 2B,C). Furthermore, we found that the level of IFN-γ^+^ T cells was markedly increased 45 days after restimulation of intestinal mucosal-associated lymphocytes with H9N2 WIV (Figure 2D). These data confirmed the ability of a mucosal vaccine to induce substantial T-cell responses after oral immunization. However, our study found that adjuvant spore immunization significantly upregulated the expression levels of the central memory T cell (Tcm) surface markers CD62L and CCR7 in blood at 7 days after primary immunization (Figure S1A,B) (*p* < 0.01). Nevertheless, no significant difference was observed at 45 days (Figure S1C,D). These results suggest that spores plus H9N2 WIV effectively induced and activated mucosal Trms, which might sustain the training of mucosal immunity.

### 3.3. Spore-Adjuvant Immunization Induces HA-Specific Trms in Intestinal Tissue

We also investigated the generation of antigen-specific Trms after mucosal immunization. To detect whether FTY720 treatment could inhibit lymphocyte circulation, FTY720 was first administered to inhibit the circulation of T cells 6 weeks after primary vaccination, as illustrated in Figure 2E. Then, mice were injected i.v. with anti-CD45–FITC antibodies 10 min prior to harvesting of blood samples and intestinal Peyer’s patches (PPs). Results first showed that the vast majority (>99.9%) of the intestinal samples failed to be stained with anti-CD45 antibodies after treatment with FTY720 (Figure S2). Moreover, microscopy observation showed that, 5 weeks after oral immunization, allophycocyanin (APC)-tetramer^+^ T cells were readily detectable in the terminal ileal region, which might contain PPs (Figure 2F). Consistently with the above results, we also found that influenza HA-specific T cells were significantly increased in H9N2 WIV plus spore-immunized mice, and this effect could be inhibited by FTY720 treatment (Figure 2G). This result suggested that H9N2 WIV plus spore-induced HA-specific T cells displayed a tendency toward tissue residency. Together, these results demonstrated that mucosal immunization with H9N2 WIV plus spore could generate influenza-specific T cells in the intestinal tract.

### 3.4. Spores Upregulate ICAM1 to Recruit and Activate DCs 

#### 3.4.1. Spores Effectively Recruit and Activate Submucosal DCs

**Figure 3:**
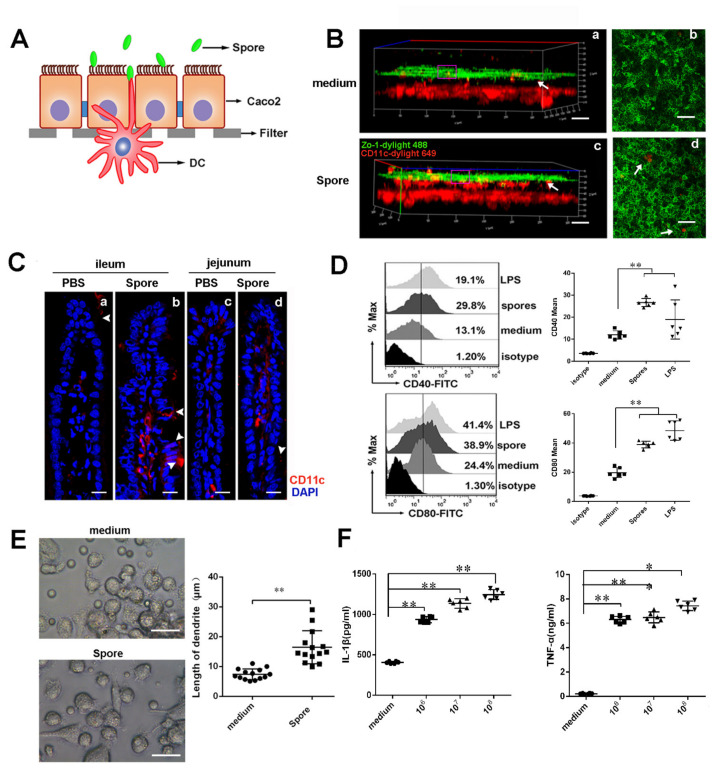
Spores activate dendritic cells in vitro and in vivo. (**A**) Schematic of the experiment used to study activation of DCs in the DC/EC coculture system. (**B**) In the coculture system, medium (**a**,**b**) and spore (**c**,**d**) were incubated on the apical side of the Caco-2 monolayer for 30 min. The filters were processed for immunofluorescence staining and observed by CLSM. A three-dimensional (3D) rendering of representative fields was obtained with the ZEN2012 software. BMDCs (CD11c, red) caused dendrites (white arrow) to creep through the tight junctions (TJs) of ECs (zo-1, green) in response to spore but not to medium. Scale bars = 50 μm. (**C**) Ligated loops of mice were injected with spore in vivo; after 30 min, intestines were isolated and then processed for immunofluorescence staining. Cryosections stained with anti-CD11c antibody (red) and 4′,6-diamidino-2-phenylindole (DAPI, blue) were observed under a confocal microscope. TEDs are indicated by arrows. Scale bars = 10 μm. (**D**) In the coculture system, DCs were stimulated for 24 h with LPS (100 ng/mL) or spores (10^6^, 10^7^, and 10^8^ CFU/mL), and the expression of surface molecules on gated viable cells was measured by flow cytometry. The phenotypic expression levels of CD40 and CD80 on DCs were analyzed by flow cytometry. (**E**) DCs were treated with medium and spore separately for 24 h, and the morphology of DC dendrites was observed by microscopy. Scale bar = 20 μm. (**F**) Secretion of interleukin (IL)-1β and TNF-α in culture supernatants was quantified by ELISA. The results are expressed as the mean ± SEM. Significant differences with the unstimulated control were tested by one-way ANOVA (*n* = 6) (* *p* < 0.05, ** *p* < 0.01).

The above data showed that spores enhanced H9N2 WIV’s ability to induce HA-specific Trms in intestinal tissue. We then investigated via which mechanism spores sustained the induction and training of Trms in the intestine. Since dendritic cells (DCs) are essential for the emergence of T-cell immunity after mucosal immunization, we hypothesized that spores might recruit and activate submucosal DCs, which would subsequently prime Trms. To investigate whether spores could recruit submucosal DCs to form transepithelial dendrites (TEDs) for viral capture, we assessed TED formation in vitro and in vivo. Firstly, we observed whether spores, but not medium, induced DCs to form TEDs across ECs at 30 min in cross-sectional images in the DC/epithelial cell (EC) coculture system (Figure 3A,B). Next, experiments with mice ligated loops showed that, 30 min after spore administration, DCs apparently gathered to the lamina propria (LP) of ECs, and their accumulation was significantly increased by treatment with spores compared to the control (Figure 3C). Moreover, we found that spores had the ability to significantly increase the expression of CD40 and CD80 (*p* < 0.01) (Figure 3D) in the coculture system, compared with the medium alone. Furthermore, we also observed that spore treatment significantly increased the length of DCs (Figure 3E). Lastly, we found that the release of proinflammatory cytokines (IL-1β and TNF-α) stimulated by spores also indicated the functional maturation of DCs (Figure 3F) (*p* < 0.01).

#### 3.4.2. Spores Upregulate ICAM1 to Recruit and Activate DCs 

**Figure 4:**
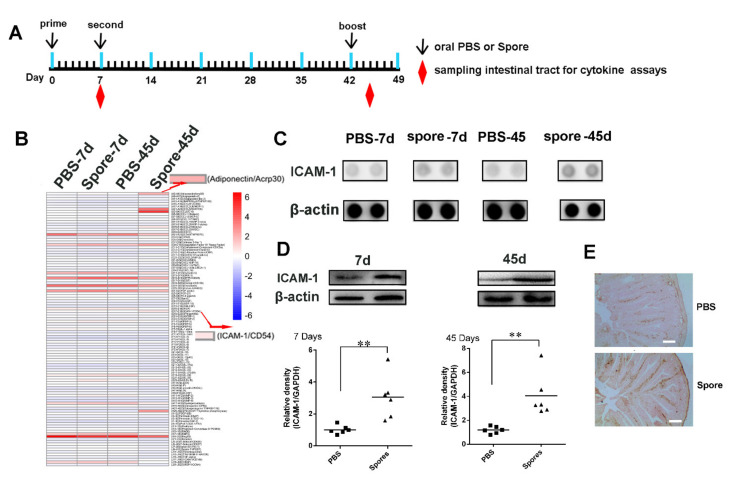
ICAM1 expression in the intestinal tract after oral administration of spore. (**A**) Schematic experimental design of oral immunization with PBS or spores and the sampling schedule of intestinal tissue and lymphocytes. (**B**) Gray value intensity was detected utilizing chemiluminescence, and membranes could be assessed for protein levels. Intensity is shown in a pseudo color scale, from low (blue) to high (red). (**C**) Mouse intestinal whole-tissue lysate was analyzed through an XL mouse antibody array. Solid black circles indicate proteins secreted by mice. (**D**) Western blot analysis revealed the time-dependent upregulation of ICAM1 in the ileum tissue following immunization with spore at 7 days and 45 days. Equal protein loading was confirmed through analysis of the housekeeping gene β-actin. (**E**) Induction of ICAM1 expression was confirmed by immunohistochemistry (IHC) staining in the ileum. Scale bar = 20 μm. Data are presented as the mean ± SEM (*n* = 6); ** *p* < 0.01.

**Figure 5:**
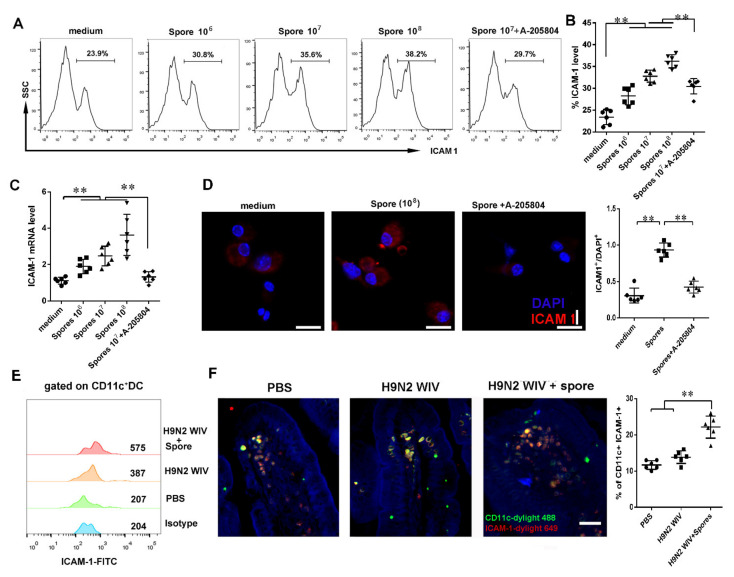
ICAM1 expression on BMDCs is increased after stimulation with spores. BMDCs were treated with spores (10^6^ CFU/mL, 10^7^ CFU/mL, and 10^8^ CFU/mL) or spores plus A-205804 (10 μM) for 24 h. (**A**,**B**) ICAM1 expression on BMDCs after spore treatment was detected by flow cytometry. (**C**) *ICAM1* mRNA expression was measured by RT-qPCR and confocal microscopy. (**D**) ICAM1 protein expression was detected by immunofluorescence after BMDCs were incubated with medium, spores, or spores plus ICAM1 inhibitor A-205804 for 24 h. Scale bar = 20 μm. (**E**) Cells were collected from the intestinal tract, and the MFI of ICAM1 gated from CD11c^+^ DCs was detected by flow cytometry. (**F**) ICAM1^+^ CD11c^+^ double-positive cells in the intestine were strongly positively stained by immune-fluorescent staining. Scale bar = 20 μm. Data are presented as the mean ± SEM (*n* = 6); ** p < 0.01.

To further reveal how spores activate intestinal submucosal DCs, we first used proteomic array profiling to analyze 110 proteins from intestinal tract lysates 7 days or 45 days after oral immunization of mice with PBS or spores (Figure 4A,B). Proteomic arrays are simple and economical multiplex assays, which allows for the measurement of more than 100 proteins in a single sample. The results showed 12 highly expressed proteins in spore-stimulated mice, while four of them were significantly increased after spore stimulation (Table S1). A further array experiment of mouse intestinal whole-tissue lysate showed that ICAM1 and adiponectin/Acrp30 were relatively upregulated in intestinal tissue after spore immunization as compared to PBS treatment (Figure 4B,C). Moreover, Western blot and immunohistochemical staining also confirmed that ICAM1 was highly expressed in spore-stimulated intestinal tissue (Figure 4D,E) (*p* < 0.01). Hence, we then focused on the functions of ICAM1 and Acrp30 in DCs. Secondly, we aimed to evaluate whether spores could promote the expression of ICAM1 on DCs. Our result showed that ICAM1 expression on DCs was significantly increased by spore treatment (10^6^, 10^7^, or 10^8^ CFU/mL) in a concentration-dependent manner (Figure 5A,B) (*p* < 0.01). DCs partially required the expression of ICAM1 induced by spores and suppressed by ICAM1 inhibitor A205804. QPCR and immunofluorescence experiments showed similar results that spore stimulation increased the levels of ICAM1 measured (Figure 5C,D) (*p* < 0.01). A previous study suggested that upregulation of ICAM1 in APCs could regulate the generation of central memory cells. We then wondered whether DCs recruited and activated by spore could migrate to the LP and then activate ICAM1 molecules to further stimulate T cells. To verify this hypothesis, we isolated mouse CD11C^+^ DCs after immunization with spores plus H9N2 WIV and found that ICAM1 was indeed upregulated with respect to immunization by H9N2 WIV alone (Figure 5E). Lastly, we found that the number of ICAM1^+^ DCs (CD11c^+^) was significantly increased in the group treated with spores plus H9N2 WIV (Figure 5F). In brief, our results suggested that ICAM1 was notably increased on DCs after spore stimulation in vitro and in vivo.

### 3.5. Spore Stimulate Acrp30 Secretion Leading to High Expression of ICAM1 

**Figure 6:**
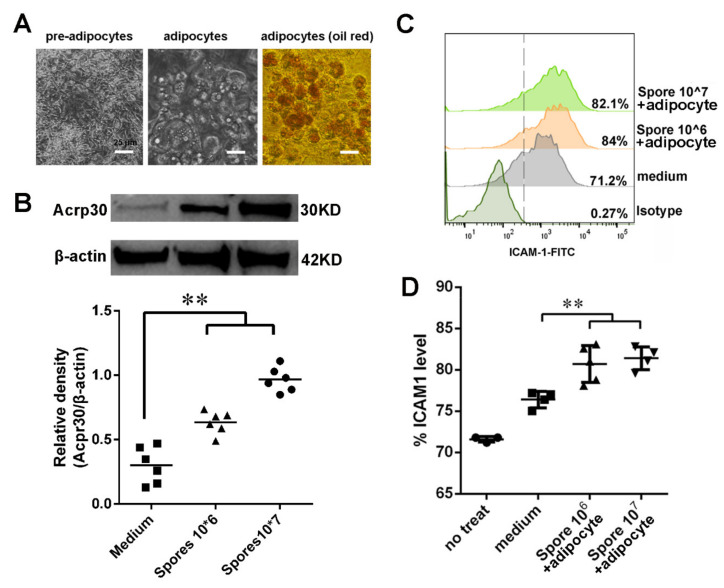
Secretion of Acrp30 from adipocytes induced by spores increased ICAM1 expression on DCs. (**A**) 3T3-L1 cells were differentiated into adipocytes according to the differentiation protocol. Phase-contrast images of 3T3-L1 cells from 0 days of induction (preadipocyte) to 10 days post induction (adipocyte). Triglyceride staining of 3T3-L1 cells was detected with Oil Red O. (**B**) Differentiated 3T3-L1 cells were treated with spores for 24 h. A Western blot assay was performed to detect the levels of adiponectin in the cells. The bar plot represents the quantification of the relative adiponectin protein levels (*n* = 6). (**C**,**D**) DCs were treated for 24 h with medium from differentiated 3T3-L1 cells treated with spores. Flow cytometry analysis was performed with anti-ICAM1–FITC staining. A representative blot is shown in the upper panel. Data are presented as the mean ± SEM (*n* = 4); ** p < 0.01. Scale bars = 25 μm.

The above data suggested that spore stimulation could increase the expression of ICAM1 to recruit and activate DCs, which could then induce the generation of HA-specific T cells. On the other hand, our proteome array data showed that both ICAM1 and Acrp30 were relatively upregulated in intestinal tissue after spore stimulation (Figure 4A). Therefore, we investigated the function of Acrp30 in DCs. Considering that Arcp30 is an adipocyte-specific secreted protein, which was highly expressed in the spore-stimulated group, we first tried to determine whether spore stimulation could lead to Arcp30 secretion by differentiated 3T3-L1 cells. Results showed that 3T3-L1 morphology changed and accumulated lipid droplets internally during the process of differentiation. Indeed, after 12 days, when the adipocytes were mature, almost the entire cell volume was stained by red oil (Figure 6A). Secondly, we found that spores at 10^6^ CFU/mL were sufficient to elicit upregulation of Acrp30. Moreover, treatment with 10^7^ CFU/mL of spores led to a twofold increase in the amount of Acrp30 protein with respect to the control without spore (Figure 6B). The extent of such a spore-induced increase in protein levels was positively associated with the spore concentration, indicating a dose-dependent effect of spores in the accumulation of Acrp30. Lastly, we wanted to evaluate whether spore-induced medium (high concentration of Acrp30 protein) had some function in DCs. Since a previous study found that Acrp30 receptor signaling in DCs can interfere with T-cell functions, we investigated whether spore-stimulated medium could increase ICAM1 expression on DCs. Interestingly, the culture supernatant from differentiated 3T3-L1 cells treated with spores or PBS could stimulate ICAM1 expression on DCs (Figure 6C,D). Collectively, our data demonstrated that spores effectively upregulated the expression of the Acrp30 and enhanced ICAM1 expression on DCs.

### 3.6. DCs Induce ICAM1-Dependent Generation of HA-Specific T Cells

**Figure 7:**
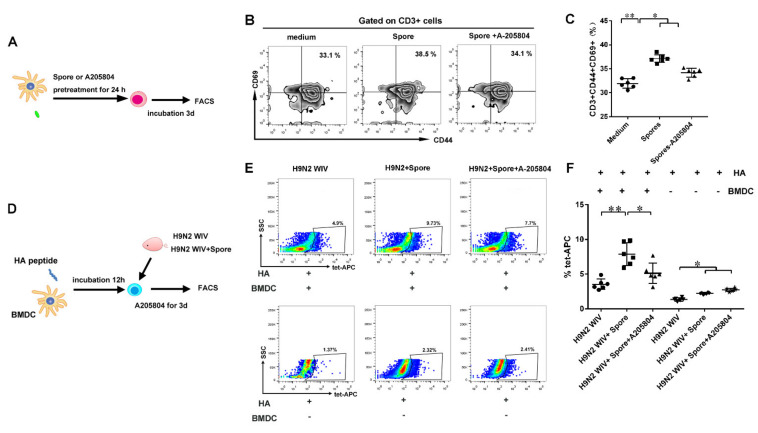
DC-promoted formation of HA-specific Trms depends on ICAM1. (**A**) DCs were pretreated with spore and/or A204804 and then coincubated with sorted CD3^+^ T cells from wild-type mice for 3 days. (**B**,**C**) Gated T cells were analyzed for the presence of the surface markers of memory cells CD44 and CD69 by FACS. (**D**) Six weeks after primary vaccination, the intestines were dissected to prepare lymphocytes. BMDCs (5 × 10^5^ cells/well) were stimulated with HA_518–526_ (IYSTVASSL) peptide (10 μL) overnight. Antigen-pulsed DCs were used as APCs to stimulate IMALs (1 × 10^6^ cells/well) for 5 days. (**E**,**F**) Frequency of APC-tetramer^+^ T cells was measured by flow cytometry. Representative flow cytometry results and graphs for statistical analysis are shown. Data are presented as the mean ± SEM (*n* = 6); * p < 0.05, ** p < 0.01.

Pretreatment with spores and/or the ICAM1 inhibitor A-205804 suppressed ICAM1 expression on DCs. At the same time, the phenotypic markers of T cells CD44 and CD69 were detected by fluorescence-activated cell sorting (FACS). As expected, flow cytometry analysis showed significantly increased levels of CD44^+^ CD69^+^ Trms upon spore treatment of DCs, whereas treatment with the ICAM1 inhibitor A-205804 suppressed the CD44^+^ CD69^+^ phenotype induced by spores (Figure 7A–C and Figure S3A) (*p* < 0.01). Meantime, we also found that both IL15 and TGF-β of BMDCs were highly expressed after spore stimulation, suggesting that intestinal Trms might be activated after spore stimulation (Figure S4).

Moreover, to assess whether HA-tetramer^+^-specific T cells persisted in the intestine at 6 weeks after immunization, antigen-loaded DCs treated or not with ICAM1 inhibitor were incubated with IMALs isolated from immunized mice at 37 °C for 3 days (Figure 7D). Flow cytometry showed that spores plus H9N2 WIV induced the generation of more HA-tetramer^+^-specific cells than H9N2 WIV alone in the presence of antigen-pulsed DCs (Figure 7E,F and Figure S3B). Taken together, these results indicated that DCs partially require the expression of ICAM1 for the generation of antigen-specific Trms, since this was impaired by the incubation of DCs with a ICAM1 inhibitor.

## 4. Discussion

Oral immunization is beneficial for eliciting mucosal immune responses against pathogens that invade their host through a mucosal surface [6]. Here, we used a mucosal immunization strategy known as “prime-boost vaccination” and dissected the multifaceted adaptive immune mechanisms triggered by mucosal immunization with spore plus H9N2 WIV (Figure 1A and Figure 2A). This strategy may represent an ideal platform for immunological protection and lead to robust antibody responses to generate long-lasting immunological memory. Indeed, this platform has numerous advantages, as spores survived well and positively stimulated DCs. Additionally, APCs such as DCs promote the generation of T cells with different fates, such as distinct populations of memory T cells, in the absence of antigen [28]. Recent studies demonstrated that potent activation signatures in macrophages and bone marrow DCs after treatment with *Bacillus subtilis* spore treatment are accompanied by increased expression levels of the maturation markers CD40 and MHC classes I and II in DCs [11]. Moreover, the recruitment of DCs, especially CD103^+^ DCs, promotes CD103 expression on immune cells and is essential for the efficient induction of Trms [29]. Subsequently, we focused on the innate immune system. Here, we performed experiments using a DC/EC coculture system and showed that spores played important roles in facilitating the delivery of DCs across intestinal mucosal barriers at an early stage of mucosal immunity and induced DC maturation, including the secretion of IL-1β. A previous study showed that IL-1β as a mucosal vaccine adjuvant led to the expression of chemokines and adhesion molecules, as well as induced local Trms, which improved the heterosubtypic immunity against influenza [30]. 

ICAM1 expression is known to play a crucial role in the proper generation of T-cell memory responses by APCs [31]. As is known, ICAM1 can act as a viral receptor, which, in response to virus stimulation, leads to increased ICAM1 expression on intestinal DCs. Since ICAM1 is also involved in the regulation of allergic reaction as an inflammatory mediator, allergens such as parasites and pollen can increase ICAM1 expression in the gut. Furthermore, ICAM1 has been proven to have a much stronger impact on the initiation of mucosal memory T cells. Thus, the difference in mucosal T-cell numbers and function between immunized animals in different groups likely reflects the response to spore-induced recruitment signals in the intestine. In this study, we identified ICAM1 as a critical regulator of DCs in inducing memory T-cell information (Figure 5). When the expression of ICAM1was inhibited, the memory T-cell phenotype markers CD44 and CD69 were observed less frequently than in the regular DC group (Figure 7). Current studies have shown that potential KLRG1^−^ CD8^+^ Trms precursors with increased expression of CD69 can be isolated from the intestine, which remain the most reliable markers for intestinal-resident T cells in mice [14,32]. A previous model predicted that ICAM1 is required to boost the priming process, likely by promoting the recruitment of naïve T cells, prolonging cell–cell interactions, facilitating cytokine signaling, and allowing the differentiation of memory T-cell precursors [18]. However, spore-induced mucosal cytokines such as IL-1β were not sufficient to induce differentiation of antigen-experienced T cells into Trms. The discovery might provide new insight into the significance of adipocyte metabolism related molecules in the regulation of ICAM1 on DCs.

In addition to ICAM1, Acro30 is another important protein highly expressed by long-term spore stimulation. As is known, the function of adipocytes is to store energy. Additionally, adipocytes have endocrine functions and can secrete a variety of adipokines such as leptin, adiponectin, and resistin [33,34]. Among them, adiponectin (Acrp30) has been demonstrated to be not only related to metabolic diseases, such as obesity, type 2 diabetes mellitus, and obstructive sleep apnea syndrome, but also involved in the regulation of immune response, such as rheumatoid arthritis and systemic lupus erythematosus [35,36,37]. In our study, we clearly demonstrated that *Bacillus subtilis* spores administrated together with H9N2 WIV could significantly activate mouse Trms cells by upregulating ICAM1 on DCs. Furthermore, we found that long-term stimulation with *Bacillus subtilis* spores hugely increased ICAM1 and ACRP30. We then investigated the function of Acrp30. As is known, Acrp30 is an important regulator of macrophage proliferation, and the receptors of Acrp30 are expressed on the surface of mononuclear cells [38,39]. DCs are not the only type of mononuclear cells, and they also have a similar function to macrophages. We then investigated whether Acrp30, in our study, could upregulate ICAM1 on DCs. Fortunately, our in vitro experiment, on one hand, found that spore stimulation highly increased the expression of Acrp30. On the other hand, we demonstrated that this spore-stimulated adipocyte supernatant significantly upregulated the expression of ICAM1 in DCs. These results confirmed our hypothesis that Acrp30 might directly induce DCs to increase the expression of ICAM1. In addition, Acrp30 may exert different functions; for example, Acrp30 enhances IL-6 production in primary human monocytes, while it downregulates TNF-α expression to inhibit the growth of myelomonocytic progenitors [40,41,42]. Moreover, in vitro experiment indicated that spore-induced Acrp30 from adipocytes promoted ICAM1 expression on DCs (Figure 6). This discovery might provide new insight into the significance of adipocyte metabolism-related molecules in the regulation of ICAM1 on DCs.

To investigate the antibodies associated with mucosal responses, we first evaluated antigen-specific antibody production in serum and mucosal fluid. We found that mice immunized with spores plus H9N2 WIV produced more specific IgG in the serum and IgA in the mucosal fluid as compared to mice treated with H9N2 WIV alone (Figure 1). A similar trend was observed for lymphocyte proliferation after recalling antigens. Overall, these findings may reflect the detection limit of our assays or at least the presence of some potential metabolites of *Bacillus subtilis* spores as active components of mucosal immune enhancement. 

**Figure 8:**
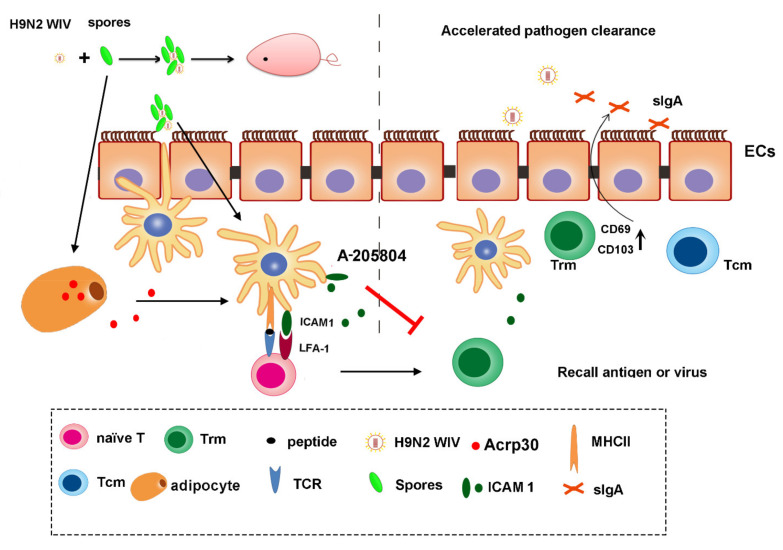
Overview of the generation of memory T-cell populations after mucosal vaccination. Upon mucosal vaccination, DCs were activated by H9N2 WIV plus spores, before migrating to the draining lymph nodes where they stimulated naïve T cells. Tcms recirculated between blood and lymphoid organs or entered peripheral tissues. Vaccination also activated DCs to express ICAM1 and cytokines, as well as LFA-1, binding to ICAM1 molecules on DCs. In addition, Acrp30 induced by the differentiation of 3T3-L1 cells into adipocytes upon spore treatment might promote ICAM1 expression on DCs. Then, ICAM1 expression on DCs could upregulate the proportion of antigen-specific Trms, which was altered after treatment with an ICAM1 inhibitor. The surface markers of Trms, such as CD69 and CD103, were upregulated after the induction of mucosal immunity. Local reactivation of mucosal Trms formation was induced by the ICAM1 molecule, which triggered DC recruitment and cytokine expression. The existence and maintenance of Trms subsets in the intestine could accelerate pathogen clearance.

In summary, in this study, spores could dramatically increase the percentage of proliferating lymphocytes, indicating either a higher frequency of memory cells or the presence of cells with a higher proliferative capacity. Along with conventional T-cell activation signatures, we also observed a striking accumulation of large CD69^+^ CD103^+^ Trms in intestinal tissue after immunization with spores plus H9N2 WIV, as schematically presented in Figure 8. Additional unknown factors necessary for the establishment of Trms may play critical roles in this process. For instance, recent vaccine studies have demonstrated that mucosal administration of antigens is important for the establishment of localized T-cell responses [14]. In an optimally immunized individual, Trms were more protective than circulating memory cells due to their location and function [43]. The fact that no Trms targeted the spores themselves may be justified by the behavior of spores as mammalian commensal agents [44], resulting in suppressed mobilization of Trms, which would lead to their clearance [45].

## Data Availability

Data is contained within the article or Supplementary Material.

## Supplementary Materials

The following are available online at https://www.mdpi.com/article/10.3390/cells10092267/s1, Figure S1: Spore plus H9N2 WIV generated abundant CD62L+CCR7+ cells during the early immunization period. Figure S2: Evaluation of tissue-resident T cells with a combination of FTY720 treatment and IV staining. Figure S3: FACS gating strategy of Trm cells and tet-APC cells. Figure S4: Detection of IL-15 and TGF-βexpression of Mice BMDCs stimulated by spore or A205804. Table S1: Proteomic array result of 110 proteins from intestinal tract lysates 7 days or 45 days after oral immunization of mice with PBS or spores. Table S2: Primer used in detection IL-15 and TGF-β expression of Mice BMDCs stimulated by spore or A205804.

## Abbreviations

BMDCs	bone marrow-derived dendritic cells	
AIV	avian influenza virus	
Tcms	central memory T cells	
Trms	tissue-resident memory T cells	
Tems	effector memory T cells	
S1P	sphingosine-1-phosphate	
TEDs	transepithelial dendrites	
EC	epithelial cell	
GM-CSF	granulocyte colony-stimulating factor	
MHC-II	major histocompatibility complex class II	
FACS	Fluorescence-Activated Cell Sorter	
Key Resources Table		
Reagent or Resource	Source	Clone
anti-mouse CD11c–APC	eBioscience	N418
anti-mouse CD40–PE	eBioscience	1C10
anti-mouse CD80–FITC	eBioscience	16-10A1
anti-mouse CD86–PE	eBioscience	GL1
anti-mouse MHC class II I-A–FITC	eBioscience	NIMR-4
anti-mouse CD3e–APC	eBioscience	145-2C11
anti-mouse CD62L–APC	Miltenyi	REA828
anti-mouse CCR7–PE	Miltenyi	REA685
recombinant murine GM-CSF	Peprotech	Cat#214-14-204G
recombinant murine IL-4	Peprotech	Cat#315-03-204G 2E7
anti-mouse CD69–PE	eBioscience	M290
anti-mouse CD103–FITC	eBioscience	XMG1.2
anti-mouse INF-γ–PE	eBioscience	IM7
anti-mouse CD44–FITC	eBioscience	EPR22161-284
rabbit anti-mouse ICAM1	Abcam	PA1-054
rabbit anti-mouse Acrp30	Thermo Fisher	4D3
rabbit anti-mouse β-actin	Bioworld	N/A
influenza HA_518–526_ (IYSTVASSL) peptide	Genscript	N/A
HA-specific (HA_518–526_)	MBL	
H-2Kd tetramer-IYSTVASSL		

## References

[1] Iqbal M., Yaqub T., Mukhtar N., Shabbir M.Z., McCauley J.W. (2013). Infectivity and Transmissibility of H9N2 Avian Influenza Virus in Chickens and Wild Terrestrial Birds. Vet. Res..

[2] Sun Y.P., Liu J.H. (2015). H9N2 Influenza Virus in China: A Cause of Concern. Protein Cell.

[3] Qu B.Q., Li X., Gao W., Sun W.K., Jin Y., Cardona C.J., Xing Z. (2012). Human Intestinal Epithelial Cells Are Susceptible to Influenza Virus Subtype H9N2. Virus Res..

[4] Wille M., Brojer C., Lundkvist A., Jarhult J.D. (2018). Alternate Routes of Influenza a Virus Infection in Mallard (*Anas Platyrhynchos*). Vet. Res..

[5] Van Splunter M., van Hoffen E., Floris-Vollenbroek E.G., Timmerman H., Lucas-van de Bos E., Meijer B., Ulfman L.H., Witteman B., Wells J.M., Brugman S. (2018). Oral Cholera Vaccination Promotes Homing of IgA(+) Memory B Cells to the Large Intestine and the Respiratory Tract. Mucosal Immunol..

[6] Luo J., Liu X.P., Xiong F.F., Gao F.X., Tan W.S. (2019). Enhancing Immune Response and Heterosubtypic Protection Ability of Inactivated H7N9 Vaccine by Using STING Agonist as a Mucosal Adjuvan. Front. Immunol..

[7] Ji E.L., Kye Y.C., Park S.M., Shim B.S., Yun C.H. (2020). Bacillus Subtilis Spores as Adjuvants Against Avian Influenza h9n2 Induce Antigen-Specific Antibody and T Cell Responses in White Leghorn Chickens. Vet. Res..

[8] Zhao G.Y., Miao Y., Guo Y., Qiu H.J., Sun S.H., Kou Z.H., Yu H., Li J.F., Chen Y., Jiang S.B. (2014). Development of a Heat-Stable and Orally Delivered Recombinant M2e-Expressing B. Subtilis Spore-Based Influenza Vaccine. Hum. Vacc. Immunother..

[9] Song M., Hong H.A., Huang J.M., Colenutt C., Khang D.D., Thi V.A.N., Park S.M., Shim B.S., Song H.H., Cheon I.S. (2012). Killed Bacillus Subtilis Spores as a Mucosal Adjuvant for an H5N1 Vaccine. Vaccine.

[10] Esparza-Gonzalez S.C., Troy A.R., Izzo A.A. (2014). Comparative Analysis of Bacillus Subtilis Spores and Monophosphoryl Lipid a As Adjuvants of Protein-Based Mycobacterium Tuberculosis-Based Vaccines: Partial Requirement for Interleukin-17a for Induction of Protective Immunity. Clin. Vaccine Immunol..

[11] Copland A., Hart P., Diogo G.R., Harris S., Paul M., Singh M., Cutting S.M., Reljic R. (2018). Mucosal Delivery of Fusion Proteins with Bacillus subtilis Spores Enhances Protection against Tuberculosis by BCG. Front. Immunol..

[12] Kim S.-H., Yang I.-Y., Kim J., Lee K.-Y., Jang Y.-S. (2015). Antimicrobial Peptide Ll-37 Promotes Antigen-Specific Immune Responses in Mice by Enhancing th17-Skewed Mucosal and Systemic Immunities. Eur. J. Immunol..

[13] Wu T., Hu Y.H., Lee Y.T., Bouchard K.R., Benechet A., Khanna K., Cauley L.S. (2014). Lung-Resident Memory CD8 T Cells (TRM) Are Indispensable for Optimal Crossprotection Against Pulmonary Virus Infection. J. Leukocyte Biol..

[14] Zens K.D., Chen J.K., Farber D.L. (2016). Vaccine-Generated Lung Tissue-Resident Memory T Cells Provide Heterosubtypic Protection to Influenza Infection. JCI Insight.

[15] Huang L.L., Wang J.L., Wang Y.H., Zhang E., Li Y.C., Yu Q.H., Yang Q. (2019). Upregulation of CD4(+) CD8 (+) Memory Cells in the Piglet Intestine Following Oral Administration of Bacillus subtilis Spores Combined with PEDV Whole Inactivated Virus. Vet. Microbiol..

[16] Qin T., Yin Y.Y., Yu Q.H., Huang L.L., Wang X.Q., Lin J., Yang Q. (2015). CpG Oligodeoxynucleotides Facilitate Delivery of Whole Inactivated H9N2 Influenza Virus via Transepithelial Dendrites of Dendritic Cells in Nasal Mucosa. J. Virol..

[17] Zheng T.T., Zhang B.H., Chen C., Ma J.Y., Meng D.Y., Huang J., Hu R., Liu X.G., Otsu K., Liu A.C. (2018). Protein Kinase p38 Alpha Signaling in Dendritic Cells Regulates Colon Inflammation and Tumorigenesis. Proc. Natl. Acad. Sci. USA.

[18] Cox M.A., Barnum S.R., Bullard D.C., Zajac A.J. (2013). ICAM-1-Dependent Tuning of Memory CD8 T-Cell Responses Following Acute Infection. Proc. Natl. Acad. Sci. USA.

[19] McNamara H.A., Cai Y., Wagle M.V., Sontani Y., Roots C.M., Miosge L.A., O’Connor J.H., Sutton H.J., Ganusov V.V., Heath W.R. (2017). Up-Regulation of LFA-1 Allows Liver-Resident Memory T Cells to Patrol and Remain in the Hepatic Sinusoids. Sci. Immunol..

[20] Dong Z., Fu S., Xu X., Yang Y., Du L., Li W., Kan S., Li Z., Zhang X., Wang L. (2014). Leptin-Mediated Regulation of ICAM-1 Is Rho/ROCK Dependent and Enhances Gastric Cancer Cell Migration. Brit. J. Cancer.

[21] Xu Z.H., Zhang R.F., Wang D.D., Qiu M.H., Feng H.C., Zhang N., Shen Q.R. (2014). Enhanced Control of Cucumber Wilt Disease by Bacillus amyloliquefaciens SQR9 by Altering the Regulation of Its DegU Phosphorylation. Appl. Environ. Microb..

[22] Geeraedts F., Goutagny N., Hornung V., Severa M., de Haan A., Pool J., Wilschut J., Fitzgerald K.A., Huckriede A. (2008). Superior Immunogenicity of Inactivated Whole Virus H5N1 Influenza Vaccine Is Primarily Controlled by Toll-Like Receptor Signalling. PLoS Pathog..

[23] Ivanov I.I., McKenzie B.S., Zhou L., Tadokoro C.E., Lepelley A., Lafaille J.J., Cua D.J., Littman D.R. (2006). The Orphan Nuclear Receptor ROR Gamma T Directs the Differentiation Program of Proinflammatory IL-17(+) T Helper Cells. Cell.

[24] Flaherty S., Reynolds J.M. (2015). Mouse Naive CD4(+) T Cell Isolation and In vitro Differentiation into T Cell Subsets. J. Vis. Exp. JoVE.

[25] Lee S.M., Do H.J., Shin M.J., Seong S.I., Hwang K.Y., Lee J.Y., Kwon O., Jin T., Chung J.H. (2013). 1-Deoxynojirimycin Isolated from a Bacillus Subtilis Stimulates Adiponectin and GLUT4 Expressions in 3T3-L1 Adipocytes. J. Microbiol. Biotechn..

[26] Gao X., Huang L.L., Zhu L.Q., Mou C.X., Hou Q.H., Yu Q.H. (2016). Inhibition of H9N2 Virus Invasion into Dendritic Cells by the S-Layer Protein from L. Acidophilus ATCC 4356. Front. Cell. Infect. Microbiol..

[27] Wein A.N., Mcmaster S.R., Takamura S., Dunbar P.R., Cartwright E.K., Hayward S.L. (2019). Cxcr6 Regulates Localization of Tissue-Resident Memory cd8 T Cells to the Airways. J. Exp. Med..

[28] Lanzavecchia A., Sallusto F. (2002). Progressive Differentiation and Selection of the Fittest in the Immune Response. Nat. Rev. Immunol..

[29] Wakim L.M., Smith J., Caminschi I., Lahoud M.H., Villadangos J.A. (2015). Antibody-Targeted Vaccination to Lung Dendritic Cells Generates Tissue-Resident Memory CD8 Tcells That Are Highly Protective Against Influenza Virus Infection. Mucosal Immunol..

[30] Lapuente D., Bonsmann M.S.G., Maaske A., Stab V., Heinecke V., Watzstedt K., Hess R., Westendorf A.M., Bayer W., Ehrhardt C. (2018). IL-1 Beta as Mucosal Vaccine Adjuvant: The Specific Induction of Tissue-Resident Memory T Cells Improves the Heterosubtypic Im-Munity Against Influenza a Viruses. Mucosal Immunol..

[31] Scholer A., Hugues S., Boissonnas A., Fetler L., Amigorena S. (2008). Intercellular Adhesion Molecule-1-Dependent Stable Interactions Between T Cells and Dendritic Cells Determine CD8(+) T Cell Memory. Immunity.

[32] Mackay L.K., Rahimpour A., Ma J.Z., Collins N., Stock A.T., Hafon M.L., Vega-Ramos J., Lauzurica P., Mueller S.N., Stefanovic T. (2013). The Developmental Pathway for CD103(+)CD8(+) Tissue-Resident Memory T Cells of Skin. Nat. Immunol..

[33] Deng T., Lyon C.J., Bergin S., Caligiuri M.A., Hsueh W.A. (2016). Obesity, Inflammation, and Cancer. Annu. Rev. Pathol. Mech..

[34] Ouchi N., Parker J.L., Lugus J.J., Walsh K. (2011). Adipokines in Inflammation and Metabolic Disease. Nat. Rev. Immunol..

[35] Daniele A., De Rosa A., Nigro E., Scudiero O., Capasso M., Masullo M., de Laurentiis G., Oriani G., Sofia M., Bianco A. (2012). Adiponectin Oligomerization State and Adiponectin Receptors Airway Expression in Chronic Obstructive Pulmonary Disease. Int. J. Biochem. Cell B.

[36] Nigro E., Daniele A., Scudiero O., Monaco M.L., Roviezzo F., D’Agostino B., Mazzarella G., Bianco A. (2015). Adiponectin in Asthma: Implications for Phenotyping. Curr. Protein Pept. Sci..

[37] Lacedonia D., Nigro E., Matera M.G., Scudiero O., Monaco M.L., Polito R., Carpagnano G.E., Barbaro M.P.F., Mazzarella G., Bianco A. (2016). Evaluation of Adiponectin Profile in Italian Patients Affected by Obstructive Sleep Apnea Syndrome. Pulm. Pharmacol. Ther..

[38] Yokota T., Oritani K., Takahashi I., Ishikawa J., Matsuyama A., Ouchi N., Kihara S., Funahashi T., Tenner A.J., Tomiyama Y. (2000). Adiponectin, a New Member of the Family of Soluble Defense Collagens, Negatively Regulates the Growth of Myelomonocytic Progenitors and the Functions of Macrophages. Blood.

[39] Pang T.T.L., Narendran P. (2008). The Distribution of Adiponectin Receptors on Human Peripheral Blood Mononuclear Cells. Ann. N. Y. Acad. Sci..

[40] Bruun J.M., Lihn A.S., Verdich C., Pedersen S.B., Toubro S., Astrup A., Richelsen B. (2003). Regulation of Adiponectin by Adipose Tissue-Derived Cytokines: In Vivo and In Vitro Investigations in Humans. Am. J. Physiol. Endoc. M.

[41] Robinson K., Prins J., Venkatesh B. (2011). Clinical Review: Adiponectin Biology and Its Role in Inflammation and Critical Illness. Crit. Care.

[42] Tsatsanis C., Zacharioudaki V., Androulidaki A., Dermitzaki E., Charalampopoulos I., Minas V., Gravanis A., Margioris A.N. (2005). Adiponectin Induces TNF-Alpha and IL-6 in Macrophages and Promotes Tolerance to Itself and other Pro-Inflammatory Stimuli. Biochem. Biophys. Res. Commun..

[43] Iijima N., Iwasaki A. (2015). Tissue Instruction for Migration and Retention of T-RM Cells. Trends Immunol..

[44] Salzman N.H., de Jong H., Paterson Y., Harmsen H.J.M., Welling G.W., Bos N.A. (2002). Analysis of 16S Libraries of Mouse Gastrointestinal Microflora Reveals a Large New Group of Mouse Intestinal Bacteria. Microbiology.

[45] Stary G., Olive A., Radovic-Moreno A.F., Gondek D., Alvarez D., Basto P.A., Perro M., Vrbanac V.D., Tager A.M., Shi J.J. (2015). A Mucosal Vaccine Against Chlamydia Trachomatis Generates Two Waves of Protective Memory T Cells. Science.

