# Supplementary material for: Bacillus subtilis Spore-Trained Dendritic Cells Enhance the Generation of Memory T Cells via ICAM1
Source: Cells. 2021 Aug 31;10(9):2267. doi: 10.3390/cells10092267 (PMC8469252; doi:10.3390/cells10092267)

S1 Fig Spore plus H9N2 WIV generated abundant CD62L$^+$CCR7$^+$ cells during the early immunization period.

The effect of spore on Tcms in blood was detected by FACS. (A -D) The frequency of Tcms (CD3$^+$ CD62L$^+$ CCR7$^+$) was detected in the blood 7 d (A and C) and 45 d (B and D) after priming immunization. A gating strategy of live cells and lymphocytes was applied, followed by gating for CD3$^+$ cells and determination of the memory cell phenotype according to CCR7 and CD62L expression. The results are expressed as mean ± SEM (n=6). *P < 0.05, **P < 0.01. One representative result three similar independent experiments is shown.

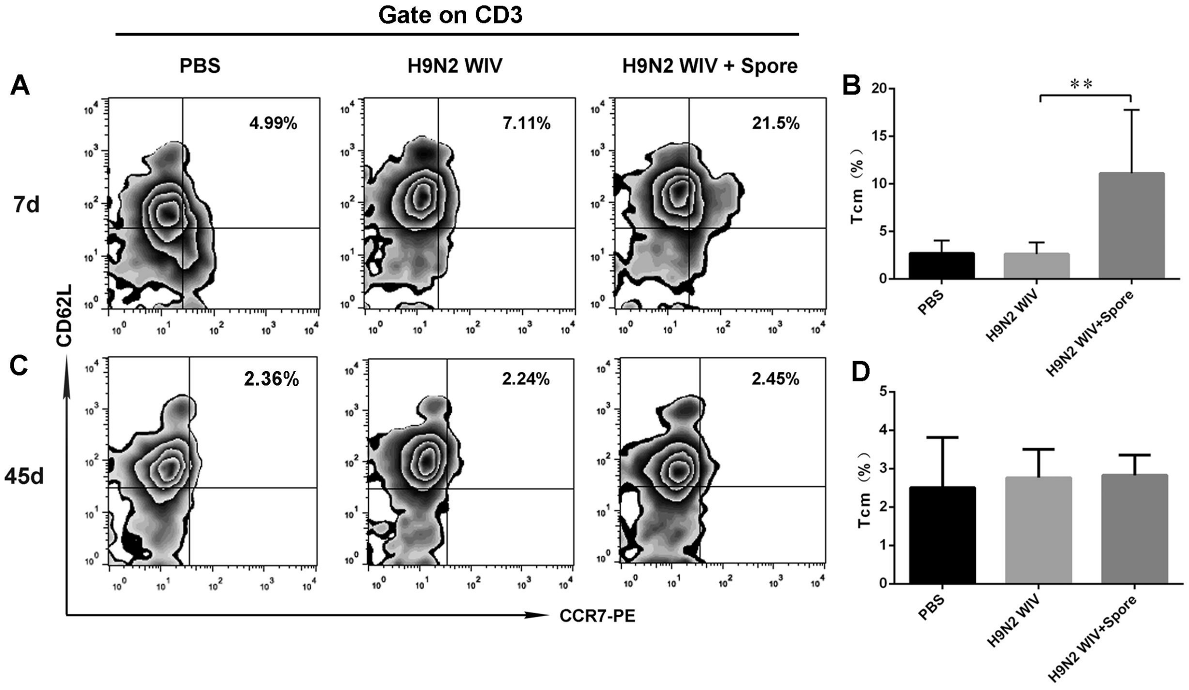

S2 Fig Evaluation of tissue-resident T cells with a combination of FTY720 treatment and IV staining.

(A and B) Vascular T cells were completely depleted by FTY720 treatment and stained by CD45-FITC I.V. staining. Schematic of vaccination, which was performed as described previously. Briefly, the immune-suppressive agent FTY720 (0.5 mg/kg/d) was administered intraperitoneally (i.p.) for 10 sequential days to deplete circulating lymphocytes six weeks after the primary vaccination. On the following day, anti-CD45 monoclonal antibody (FITC-labeled, 2.5 μg/mouse) was injected into the orbital vein to stain vascular leukocytes (IV staining) 10 min before euthanasia. Intestinal and peripheral blood leukocytes were collected to validate the efficacy of FTY720 administration and IV staining. The results are expressed as mean ± SEM (n=6). **P* < 0.05, ***P* < 0.01. One representative result three similar independent experiments is shown.

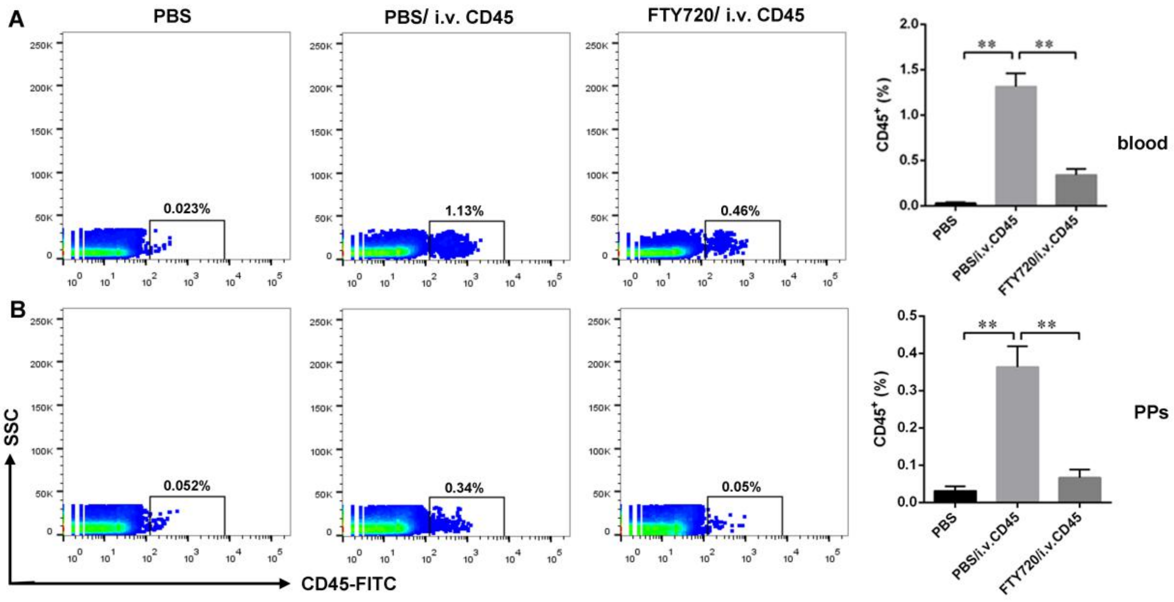

S3 Fig. FACS gating strategy of Trm cells and tet-APC cells

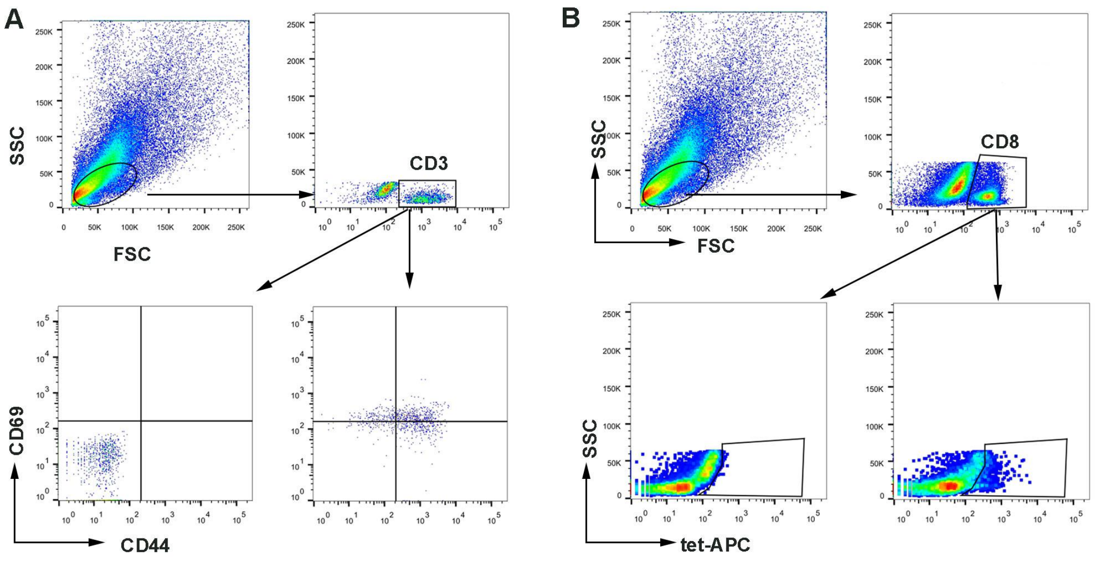

S4 Fig. Detection of IL-15 and TGF- β expression of Mice BMDCs stimulated by spore or A205804. Results are expressed as mean ± SEM (n=6). $**P < 0.01$. $***P < 0.001$. One representative result three similar independent experiments is shown.

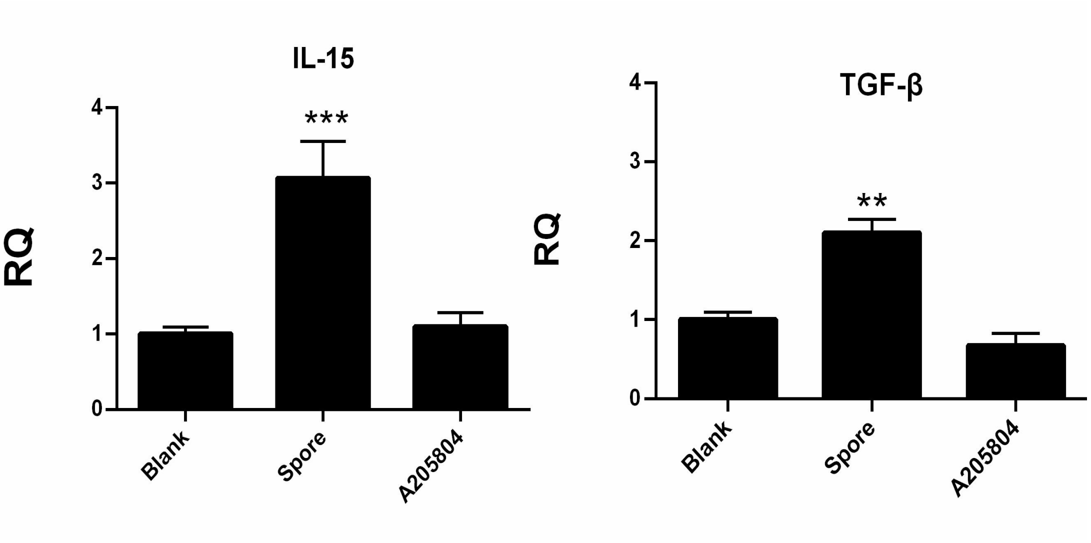

Supplement: Supplementary file 1 [file cells-10-02267-s001.zip › cells-1270598-supplementary.pdf]
